# Elucidating the Innate Immunological Effects of Mild Magnetic Hyperthermia on U87 Human Glioblastoma Cells: An In Vitro Study

**DOI:** 10.3390/pharmaceutics13101668

**Published:** 2021-10-12

**Authors:** Stefano Persano, Francesco Vicini, Alessandro Poggi, Jordi Leonardo Castrillo Fernandez, Giusy Maria Rita Rizzo, Helena Gavilán, Niccolo Silvestri, Teresa Pellegrino

**Affiliations:** 1 Nanomaterials for Biomedical Applications Department, Istituto Italiano di Tecnologia (IIT), via Morego 30, 16163 Genoa, Italy; vicini_francesco@libero.it (F.V.); giusy.rizzo@iit.it (G.M.R.R.); helena.gavilan@iit.it (H.G.); Niccolo.Silvestri@iit.it (N.S.); 2 Molecular Oncology and Angiogenesis Unit, IRCCS Ospedale Policlinico San Martino, 16132 Genoa, Italy; alessandro.poggi@hsanmartino.it (A.P.); leonardo.castrillo@hsanmartino.it (J.L.C.F.)

**Keywords:** glioblastoma, immunogenic cell death, innate immunity, natural killer, macrophages, magnetic hyperthermia

## Abstract

Cancer immunotherapies have been approved as standard second-line or in some cases even as first-line treatment for a wide range of cancers. However, immunotherapy has not shown clinically relevant success in glioblastoma (GBM). This is principally due to the brain’s “immune-privileged” status and the peculiar tumor microenvironment (TME) of GBM characterized by a lack of tumor-infiltrating lymphocytes and the establishment of immunosuppressive mechanisms. Herein, we explore a local mild thermal treatment, generated via cubic-shaped iron oxide magnetic nanoparticles (size ~17 nm) when exposed to an external alternating magnetic field (AMF), to induce immunogenic cell death (ICD) in U87 glioblastoma cells. In accordance with what has been observed with other tumor types, we found that mild magnetic hyperthermia (MHT) modulates the immunological profile of U87 glioblastoma cells by inducing stress-associated signals leading to enhanced phagocytosis and killing of U87 cells by macrophages. At the same time, we demonstrated that mild magnetic hyperthermia on U87 cells has a modulatory effect on the expression of inhibitory and activating NK cell ligands. Interestingly, this alteration in the expression of NK ligands in U87 cells upon MHT treatment increased their susceptibility to NK cell killing and enhanced NK cell functionality. The overall findings demonstrate that mild MHT stimulates ICD and sensitizes GBM cells to NK-mediated killing by inducing the upregulation of specific stress ligands, providing a novel immunotherapeutic approach for GBM treatment, with potential to synergize with existing NK cell-based therapies thus improving their therapeutic outcomes.

## 1. Introduction

Glioblastoma (GBM) is the most common type of primary malignant brain tumor and is one of the most aggressive and lethal forms of cancer, with a median survival rate of 12–15 months following diagnosis [1]. The current standard treatment protocol, involving maximal surgical resection of the tumor, followed by concomitant administration of radiotherapy and chemotherapy, the latter mostly with temozolomide (TMZ), has not produced a satisfactory life-extension, and indeed, less than 5% of patients diagnosed with GBM survive for more than 5 years [2].

In the last decade, immunotherapy has emerged as a promising therapeutic regimen for cancer therapy, showing great success in the treatment of numerous cancers, including melanoma, lung, breast, colorectal and kidney cancer, and a number of immunotherapy protocols have been approved by the Food and Drug Administration (FDA) for clinical use [3,4,5,6,7,8,9]. Despite the encouraging results achieved by cancer immunotherapy in many lymphomas and solid tumors, the outcomes in GBM patients have been rather disappointing [10]. The establishment of an immunosuppressive tumor microenvironment (TME) and the presence of the blood-brain barrier (BBB), rendering GBM an “immune-privileged” site, have been widely recognized as the main cause of this failure [11,12,13].

Brain tumor cells can directly inhibit immune responses by regulating the expression of specific surface proteins, including programmed death-ligand 1 (PD-L1), cluster of differentiation 47 (CD47), human leukocyte antigen (HLA) molecules and natural killer group 2 member D (NKG2D) ligands, or indirectly through the secretion of anti-inflammatory cytokines or chemokines promoting the recruitment of immunosuppressive cells such as tumor-associated macrophages (TAM) and myeloid-derived suppressor cells (MDSC), which constitute up to 50% of the glioma mass [14].

Most cancer immunotherapy treatments have focused on unleashing antigen-specific immune responses, driven mostly by CD8^+^ T cells [4]. The key role played by effector T cells in mediating antitumor responses has been corroborated by the positive correlation existing between tumor infiltrating T cells and prognostic outcomes [15,16,17]. However, it has been demonstrated that the onset and maintenance of CD8^+^ T cell-mediated responses and the generation of long-term immunity against tumors relies on the efficient activation of the innate immune system [18,19,20]. Additionally, innate immune cells not only sense cancer cells and initiate adaptive immune responses, but they can also exert an effector response through specific mechanisms, such as phagocytosis for macrophages and natural cytotoxicity for natural killer (NK) cells [21,22]. The enormous potential of innate immunity in GBM has been underlined by multiple studies showing that NK cells represent the most abundant intratumoral lymphocyte population and that GBM cells may be particularly vulnerable to innate effector lymphocytes [23,24,25,26,27,28]. The relevant role of innate immunity in cancer therapy is further supported by recent findings that have confirmed the existence of a subpopulation of NK cells which exhibit antigen or non-antigen specific memory-like functions, revealing that NK cells can possess features analogous to adaptive immunity [29,30,31,32].

The recognition of cancer cells by effector innate immune cells occurs via specific receptors like NKG2D, which can detect molecular alterations, such as cell surface expression of stress-inducible molecules (e.g., MHC class I chain-related proteins A and B (MICA/B) and UL16 binding proteins (ULBPs)) on target cells [33]. Innate immune cells also participate to effector responses via antibody-dependent cellular phagocytosis (ADCP) or antibody-dependent cellular cytotoxicity (ADCC) [34,35].

The importance of innate immunity is turning out to be particularly relevant in those tumors, such as GBM, that are characterized by low mutation burden, downregulation or loss of the antigen presentation, T cell dysfunction and low number of tumor-infiltrating T cells, which can limit the utility of immunotherapeutic strategies directed to promote an antigen-specific T cell response (e.g., anticancer vaccines) [13,36].

In this context, therapeutic approaches aiming to sensitize GBM cells to the recognition and killing by innate immune cells represent an effective strategy to improve the outcomes of immunotherapy.

Immunogenic cell death (ICD)-inducing strategies are a convenient mode to achieve simultaneous activation of innate and adaptive immunity by promoting the expression and exposition of stress-associated molecules and the release of tumor antigens normally hidden within cancer cells [37,38]. ICD is accompanied by the exposure and release of numerous damage-associated molecular patterns (DAMPs), including calreticulin (CTR), heat shock proteins (HSPs) and high mobility group box 1 (HMGB1). CRT exposed on the surface of dying cancer cells promotes the engulfment of dying cancer cells by antigen-presenting cells (APCs), whereas HSPs and HMGB1 bind to TLR4, enhancing APC activation and antigen cross-presentation [37,38,39]. Ultimately, ICD serves as a source of both antigens and adjuvanting molecules to activate APCs and promote antitumor immunity, providing an opportunity for a novel treatment known as “in situ vaccination” [40,41,42].

Different treatments have been identified as ICD inducers, including chemotherapy, radiotherapy and hyperthermia (HT) [43,44,45,46,47,48]. Among all, HT presents unique advantages over other ICD inducers, such as minimal invasiveness, inferior mutagenic potential and systemic toxicity and improved tumor specificity [49,50]. All this is particularly true when heat is selectively and locally applied at the tumor site, sparing the surrounding healthy tissues or other organs. This is the case of magnetic hyperthermia (MHT), which relies on the use of magnetic nanoparticles to remotely induce local heat when an alternating magnetic field (AMF) is externally applied [51,52]. Moreover, in comparison to conventional HT treatments, coupling plasmonic nanoparticles with laser light as the external energy source, MHT exhibits superior penetration with no tissue-depth attenuation of the magnetic responsiveness of magnetic nanoparticles under magnetic field conditions (100 kHz and24 kA/m) that are clinically safe for patients, rendering MHT suitable for effective heating of deep-seated tumors [53].

Although MHT has been largely investigated as an ICD inducer, the focus of these studies has been mainly toward its ability to prime a cytotoxic T lymphocyte (CTL)-dependent immune response against cancer cells, while the role played by innate effector cells in mediating ICD-induced responses has not been fully characterized [48,54,55,56]. Indeed, ICD-associated DAMPs not only favor the recruitment and activation of APCs but many of them, including HSPs, HMGB1 and NKG2D ligands, have been also demonstrated to engage receptors expressed on NK cells, potentially sensitizing tumor cells to lysis by NK cells [56,57]. Prior reports support that when locally applied, MHT can also have a direct effect on tumor-resident immune cells by promoting a decrease of MDSCs and TAM re-polarization from the M2 to M1 phenotype [48,58,59]. Upon activation, macrophages and NK cells do not only initiate and shape adaptive immune responses and function as cytokine producers, but they can also act as effector cells and be directly responsible for tumor cell elimination [59,60].

**Figure 1:**
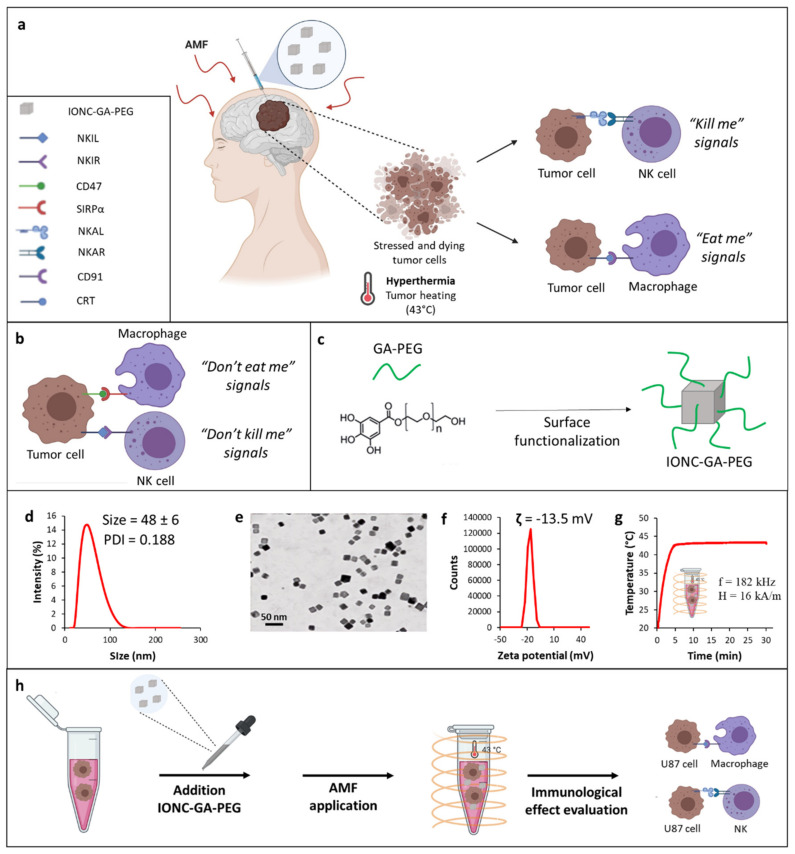
Schematic representation of the innate immunological effect of mild MHT (43 °C) on tumor cells (**a**). Tumor cells express on their surface anti−phagocytic molecules (“don’t eat me signal”), such as CD47, whereby binding to SIRP−α receptor on macrophages impedes macrophage phagocytosis of tumor cells (**b**). Tumor cells can also evade NK cell-mediated killing by increasing the expression of NK inhibitory ligands (NKILs) recognized by specific receptors (NKIRs) on NK cells and downregulating the expression of NK activating ligands (NKALs) responsible for inducing the activation of a receptor−mediated NK killing of tumor cells by engaging specific NK activating receptors (NKARs) on NK cells (**b**). All this results in a “don’t kill me” signal that allows evasion of NK cell immune responses. Mild MHT can revert the TIME thus rendering tumor cells more susceptible to innate immune effectors (macrophages and NK cells) by enhancing the expression of ICD−associated markers, such as CRT engaging the CD91 receptor on macrophages, and concomitantly upregulating NKALs and downregulating NKILs (**a**). Illustration of the functionalization of IONCs with GA−PEG polymer (**c**). Characterization of hydrodynamic size and PDI of IONC-GA-PEG via dynamic light scattering (DLS) (**d**). Representative TEM image of the IONC core coated by GA−PEG polymer (IONC−GA−PEG) (**e**). Zeta potential of IONC−GA−PEG (**f**). Temperature vs time heat profile of IONC−GA−PEG used as heat mediators to reach the desired temperature of 43 °C under an AMF at a frequency, *f*, of 182 kHz and field intensity, H, of 16 kA/m (**g**). A schematic illustration of the step-by-step in vitro study (**h**).

Here, the capacity of mild MHT (43 °C) to induce ICD, and thereby increase GBM cells’ susceptibility to innate effector cells, was evaluated on U87 GBM cells grown as 3D spheroids (Figure 1a,b). The study was conducted using iron oxide nanocubes (IONCs) with a magnetic core size of approximately 17 ± 2 nm and functionalized with gallic acid (GA)-polyethylene glycol (PEG) polymer to improve the colloidal stability of the resulting IONC-GA-PEG, thus preserving their heating properties in biological environments. 

We believe that this work offers a new rationale and design considerations for the development of novel nanotechnology-based treatments directed at eliciting both innate and adaptive immunity, for a more robust and effective antitumor immune response.

## 2. Results

### 2.1. Synthesis and Characterization of GA-PEG-Coated IONCs

The synthesis of highly monodispersed IONCs with a size of 17 ± 2 nm coated by surfactant molecules (oleic acid) was conducted according to the patented procedure [61]. Gallic polyethylene glycol (M_r_ ~1500) (GA-PEG) was chosen as a water transfer ligand for the IONCs (Figure 1c). We adapted a well-established protocol from our lab for the synthesis of GA-PEG and its further use for the water transfer of the IONCs, achieving the desired well-soluble IONC-GA-PEG in aqueous media [62].

The success of the water transfer process was also indicated by the monomodal hydrodynamic size distribution, with a main peak at 48 ± 6 nm and a polydispersity index (PDI) of 0.188, obtained via dynamic light scattering (DLS) (Figure 1d). The synthesized IONCs-GA-PEG were further characterized using transmission electron microscopy (TEM) analysis, which confirmed the production of monodispersed nanocubes separated from each other and without the presence of aggregates (Figure 1e and Figure S1 in the Supplementary Materials). The overall charge of IONCs grafted with GA-PEG molecules determined via zeta potential was slightly negative (−13.5 mV) (Figure 1f). 

The heating properties of IONCs were measured using calorimetric measurements when exposing the colloidal solution of IONCs (2 g_[Fe]_/L) to an external AMF. SAR values of the PEGylated IONCs were measured at different conditions (frequencies: 100 or 300 kHz; amplitudes: 12, 16 or 24 kA/m), confirming its high SAR values at different frequencies and field amplitudes of safe clinical use (Figure S2 in the Supplementary Materials).

It is relevant to highlight that PEGylated IONCs were able to increase the temperature media at a target therapeutic temperature of 43 °C within the initial 5 min, at a low IONC dose (2 g_[Fe]_/L) and under field conditions considered clinically safe (magnetic field intensity, H, of 16 kA/m and at a frequency, *f*, of 182 kHz) (Figure 1g).

To evaluate the biocompatibility of PEG-functionalized IONCs, an in vitro viability assay using the cell counting kit-8 (CCK-8) was conducted on human U87 glioblastoma cells (Figure S3 in the Supplementary Materials). Control groups that received either no treatment or that were exposed to AMF or IONCs alone did not show any significant variation of cell viability at 24 and 48 h. A cytotoxicity effect was observed only when U87 cells incubated with IONCs were subjected to three cycles of AMF, showing a reduction of cell viability up to more than 60% (Figure S3 in the Supplementary Materials). Taken together, these results clearly indicate that GA-PEG-capped IONCs possess optimal colloidal stability, high heat transfer efficiency and low toxicity profile in the absence of MHT actuation. Thus, IONC-GA-PEG represents an excellent heating mediator for the development of magnetic nanoparticle-mediated hyperthermia therapies.

Next, these nanoparticles were used for in vitro studies with U87 GBM cells to evaluate the immunological effect of mild MHT. A schematic of the overall study performed in U87 cells is shown in Figure 1h.

### 2.2. Mild MHT Induces Apoptosis and Impacts Tumor-Initiating and Migratory Abilities of U87 Cells

Mild hyperthermia has been shown to be effective at inducing ICD in many types of cancer cells, including glioblastoma cells [48,53,54,55,63]. ICD is a form of cell death characterized by the exposition and/or release of various DAMPs, which stimulate immune cell recruitment and activation by engaging specific receptors on the surface of innate immune cells [37]. Cell death can occur through numerous regulated mechanisms defined by specific molecular events and physiological effects. Secondary necrosis, in contrast to apoptosis or primary necrosis, is considered to be more immunogenic, as it can initiate pro-inflammatory processes leading to the activation of innate and adaptive immune responses [64].

**Figure 2:**
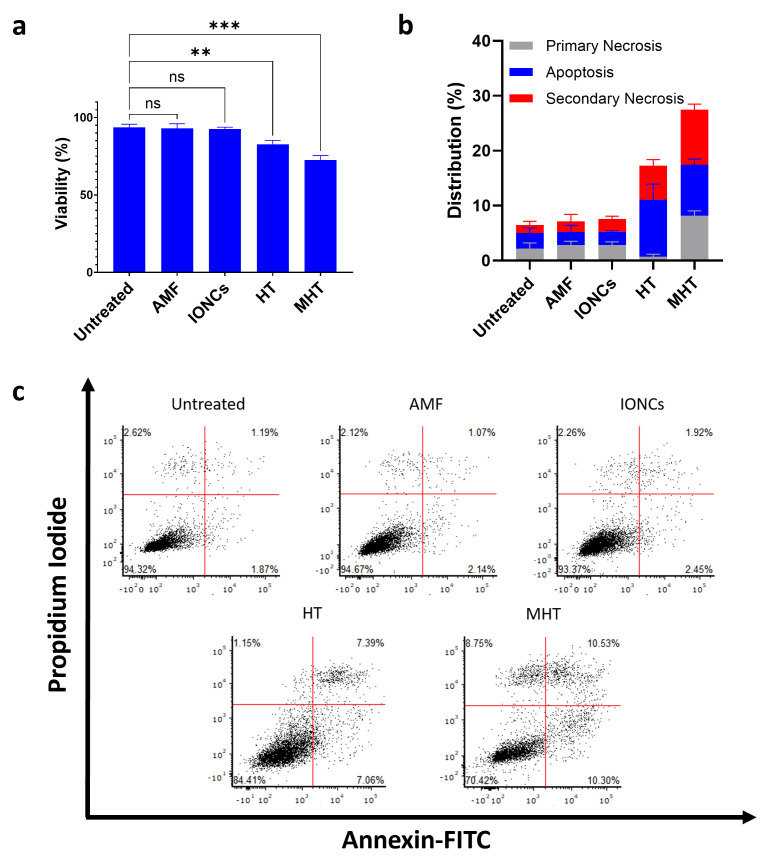
Evaluation of the ability of mild MHT to induce cell death in U87 cells measured using Annexin V/PI assay. MHT induces reduction of cell viability in U87 (**a**). After being exposed to mild MHT (3 cycles of 30 min at 43 °C), U87 cells were left to rest for 24 h and then stained with annexin-V-FITC and PI. Frequency of apoptotic (Annexin V^+^/PI^−^) (blue bars), primary necrotic (Annexin V^-^/PI^+^) (grey bars) and secondary necrotic (Annexin V^+^/PI^+^) (red bars) U87 cells were determined via flow cytometry (**b**,**c**). Data are represented as mean ± SD of three independent experiments (*n* = 3) (**b**). Representative dot plots of Annexin V/PI staining of U87 cells treated with AMF only (AMF), IONCs only (IONCs), heat bath at 43 °C (HT) or IONCs + AMF (MHT) (**c**). Statistical significance was determined with a two−tail unpaired student’s *t* test (** 0.001 < *p* < 0.01; *** *p* < 0.001; n.s. = not significant).

**Figure 3:**
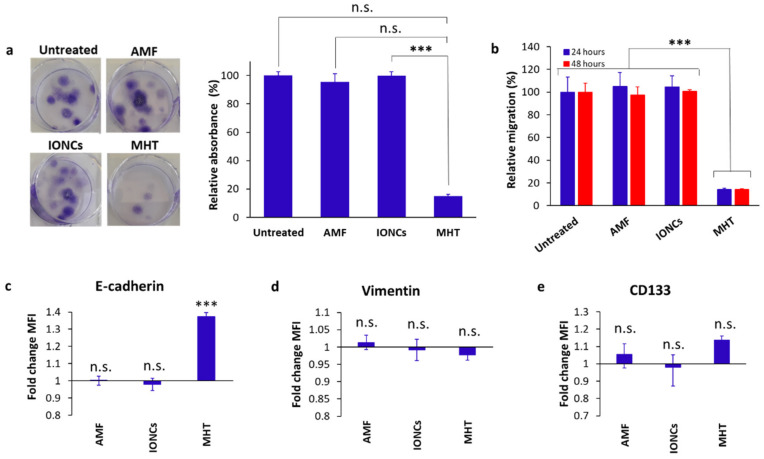
Evaluation of the clonogenic (**a**) and migration (**b**) activity, surface expression of EMT (**c**,**d**) and CSC markers (**e**) in U87 cells in response to treatment with IONCs and mild MHT (3 cycles of 30 min at 43 °C). Clonogenicity was evaluated in U87 cells after treatment with AMF, IONCs and MHT (**a**). Data are shown as representative images of stained colony with crystal violet and relative absorbance at 595 nm. A transwell assay was used to determine the migration and invasion ability of U87 cells after exposition to different treatments such as AMF only (AMF), IONCs only (IONCs) and IONCs + AMF (MHT) (**b**). The results are represented as relative percentage compared to untreated cells (**d**). Error bars indicate ± SD calculated from three independent experiments (*n* = 3). Surface expression of E-cadherin, Vimentin and CD133 was measured via flow cytometry at 24 h post-treatment with AMF, IONCs and MHT, and data reported relative to untreated controls (**c**–**e**). Data are represented as mean ± SD of three independent experiments (*n* = 3). Statistical analysis was conducted with a two−tail unpaired student’s *t* test (*** *p* < 0.001; n.s. = not significant).

In order to ascertain the ability of MHT to evoke ICD in GBM, U87 spheroids were treated with mild MHT (43 °C) and subsequently incubated at 37 °C for 24 h in serum-free medium. Cell viability in cancer cells was then evaluated via Annexin V/PI staining followed by flow cytometric analysis (Figure 2). The results showed that MHT treatment caused a reduction in cell viability of approximately 30% in U87 cells at 24 h post-treatment (Figure 2a). Interestingly, conversely to what was observed in tumor cells treated with classical hyperthermia (HT), carried out at the constant temperature of 43 °C using a thermomixer, a higher number of Annexin V/PI double positive cells (secondary necrosis) were detected at 24 h after treatment with MHT (Figure 2b,c). The cytotoxic effect of MHT in U87 cells was also confirmed with a colony-forming assay (Figure 3a) and further characterized via flow cytometric analysis of the cell cycle (Figure S4, Supplementary Materials). We found that mild MHT strongly inhibits cell proliferation in U87 cells as a consequence of an arrest of the cell cycle in the G2/M phase as evinced by the accumulation of cells in the S and G2/M phase (Figure S4 in the Supplementary Materials). 

Tumor cell migration and epithelial-mesenchymal transition (EMT) are key mechanisms that facilitate tumor progression by supporting metastasis formation, cancer stem cell (CSC) generation and drug resistance [11,16].

A transwell assay was set up to evaluate the effect of mild MHT on cell migration. Our findings showed that mild MHT strongly suppressed U87 tumor cell migration (up to ten-fold) at 24- and 48-h post-treatment (Figure 3b). Flow cytometric analysis of MHT-treated cells revealed that this observation was accompanied by increased ectopic levels of E-cadherin (Figure 3c), whereas no remarkable changes were found in the surface expression level of both CD133 and vimentin in response to treatment with MHT (Figure 3d,e). The increment in the levels of E-cadherin found in MHT-treated cells are in line with the reduced migration ability seen in U87 cells after treatment with mild MHT, since cancer cells with an epithelial phenotype are commonly characterized by a lower migration rate than those with a mesenchymal phenotype. 

### 2.3. Mild MHT Induces ICD in U87 Cells and Enhances Macrophages’ Antitumoral Functions

Cells undergoing apoptosis exhibit changes involving the exposure and release of DAMPs, such as calreticulin (CRT), HSPs, HMGB1 and other molecules, which act as danger signals to induce ICD that evoke systemic antitumor immunity.

**Figure 4:**
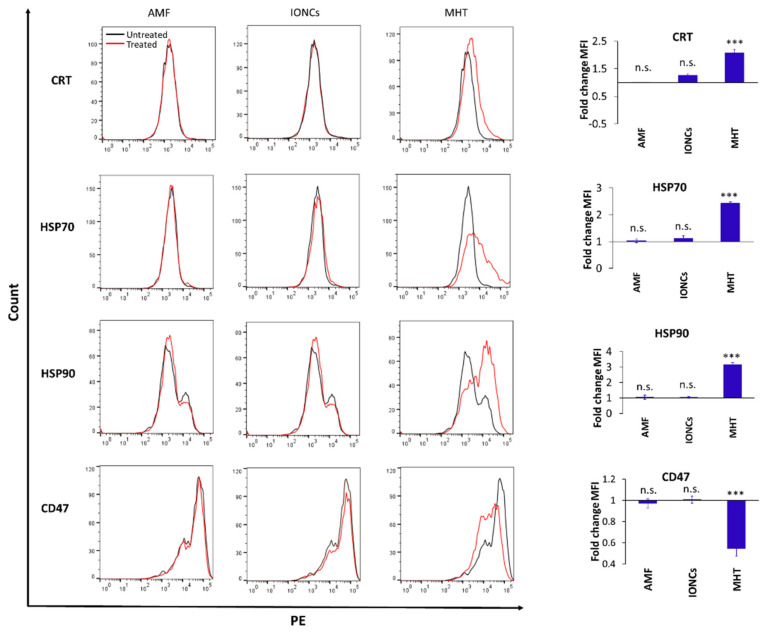
Flow cytometric evaluation of changes in surface expression of ICD-associated markers at 24 h post-treatment: CRT, HSP70, HSP90 and CD47 on U87 cells exposed to different treatments including AMF only (AMF), IONCs only (IONCs) and IONCs + AMF (MHT) over untreated cells. Data are presented as representative histograms and average fold change (mean ± SEM) of the mean fluorescence intensity (MFI) of three independent experiments (*n* = 3). Statistical analysis was conducted via two−tailed unpaired student’s *t* test (*** *p* < 0.001; n.s. = not significant).

To investigate whether MHT can act as an ICD inducer in U87 cells, MHT-treated cancer cells were analyzed for the expression and secretion of ICD-associated molecules. After treatment, we observed an increase in HMGB1 secretion (Figure S5a, Supplementary Materials), as well as in the expression of the endoplasmic reticulum (ER) chaperone protein, CRT and the cytoplasmic chaperone proteins HSP70 and HSP90 on the surface of U87 cells grown in 3D conditions (Figure 4). 

Similarly, the expression of CD47 was downregulated in MHT-treated cells compared to the control groups (Figure 4). In all cases, the expression was assessed via flow cytometry 24 h after exposure to the treatments in cells cultured as 3D spheroids.

**Figure 5:**
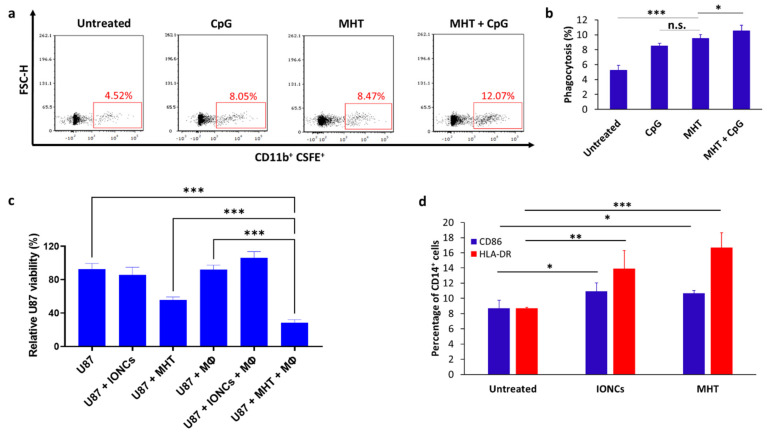
Representative dot plots showing phagocytosis of MHT−treated U87 cells by THP−1−derived macrophages (Mϕ) after 2 h of coculture at a 1:1 effector: target (E:T) ratio (**a**). Flow cytometric quantification of phagocytosis rates by THP−1−derived Mϕ towards untreated or MHT−treated U87 in the presence or absence of TLR9 agonist CpG (**b**). Percentage of phagocytosis was determined by the percentage of CSFE^+^ cells within CD11b^+^ Mϕ cell gate (double positive). Tumor cell killing by macrophages was evaluated via crystal violet staining after 24 h of co−culture with untreated or treated U87 cells with IONCs only (U87 + IONCs) or with IONCs and MHT (U87 + MHT) (**c**). Activation of THP−1−derived macrophages was evaluated via analysis of surface expression of CD86 (blue bars) and HLA−DR (red bars) after 48 of co−incubation with untreated U87 cells, U87 cells exposed to IONCs (IONCs) and MHT-treated U87 cells (MHT) (**d**). All data are shown as mean ± SD. Statistical analysis was conducted via two−tailed unpaired student’s *t* test (* 0.01 < *p* < 0.05; ** 0.001 < *p* < 0.01; *** *p* < 0.001; n.s. = not significant).

These changes in the expression of DAMPs, induced by treatment with MHT, can render cancer cells recognizable to macrophages through both downregulation of “don’t eat me” (CD47) and upregulation of “eat me” CRT signals. Thus, we evaluated in vitro the phagocytosis of MHT-treated U87 cells by THP-1 macrophages (Figure 5a,b). U87 cells exposed to mild MHT showed higher engulfment by macrophages, compared to untreated cells (Figure 5b). Additionally, in agreement with previously published studies, phagocytosis of MHT-treated U87 cells by macrophages was further enhanced in the presence of the toll-like receptor 9 (TLR9)-agonist cytosine-phosphorothioate-guanine oligodeoxynucleotide (CpG-ODN, 1 µg/mL). 

We further evaluated the ability of U87 cells undergoing ICD in response to mild MHT to induce the activation of macrophages. Consistent with MHT-induced expression of DAMPs, co-culture of THP-1-derived macrophages with MHT-treated U87 cells resulted in the upregulation of CD86 and HLA-DR (Figure 5d), both markers of APC maturation. Finally, we also demonstrated that pretreatment with mild MHT sensitizes U87 GBM cells to the killing mediated by macrophages (Figure 5c). Indeed, the viability of MHT-pretreated U87 cells was further reduced when they were co-cultured with macrophages (*p* < 0.001), while only a modest macrophage-mediated killing was observed when THP-1 macrophages were co-cultured with untreated control cells or cancer cells exposed only to IONCs (Figure 5c). Together, these in vitro results indicate that treatment with MHT can sensitize U87 glioblastoma cells to the killing mediated by macrophages, besides promoting their activation.

The PD-1/PD-L1 axis represents another immune checkpoint pathway harnessed by malignant cells to evade antitumor immune responses [65]. While initially it was thought that its role was only restricted to T cells, recent evidence pointed out that the PD-1/PD-L1 signaling pathway also participates in negatively controlling innate immune effectors [65,66]. Given the importance of PD-L1 in tumor immune evasion, we decided to assess the effect of mild MHT on PD-L1 expression in U87 cells via flow cytometry (Figure S3b,c in the Supplementary Materials). We found that mild MHT modestly downregulated PD-L1 expression on the surface of GBM cells at 24 h after treatment, whereas the exposition to AMF or IONCs did not produce any significant changes in the surface levels of PD-L1 (Figure S5b in the Supplementary Materials).

### 2.4. Mild MHT Alters the Expression of NK Cell-Activating and Inhibitory Ligands on U87 Cells

**Figure 6:**
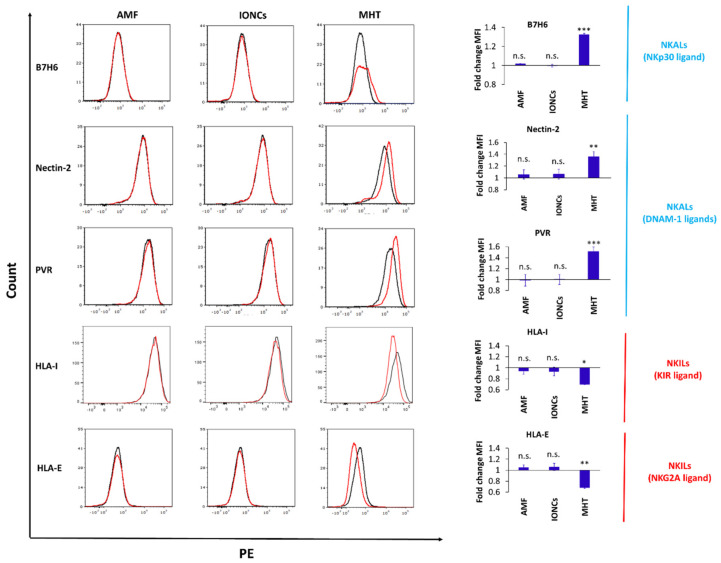
Flow cytometric evaluation of changes in surface expression of NK ligands (Nectin−2, PVR, HLA−I and HLA−E) with NK activating (NKALs) or inhibitory (NKILs) functions in U87 cells exposed to different treatments including AMF only (AMF), IONCs only (IONCs) and IONCs + AMF (MHT) compared to untreated cells at 24 h post−treatment. Data are shown as representative histograms and expressed as average fold change (mean ± SD) of the mean fluorescence intensity (MFI) of three independent experiments (*n* = 3). Statistical analysis was conducted via two−tailed unpaired student’s *t* test (* 0.01 < *p* < 0.05; ** 0.001 < *p* < 0.01; *** *p* < 0.001; n.s. = not significant).

In line with previously published works, we showed that MHT triggers the upregulation of DAMPs. Despite the role of MHT in inducing DAMPs having been already explored, its effect in modulating the expression of some stress-induced ligands and inhibitory NK-ligands on GBM cells remains to be characterized. Therefore, we investigated whether mild MHT could modulate the expression of NK cell ligands such as MICA, ULBP-1, ULBP-2 (NKG2D ligands); Nectin-2, PVR (DNAM-1 ligands); B7H6 (NKp30 ligand); HLA class I (HLA-I or HLA-ABC) and HLA-E on GBM cells. Flow cytometric analysis revealed an increase in the expression of NKp30 and DNAM-1 ligands B7H6, Nectin-2 and PVR (Figure 6) and a slight reduction in the levels of NKG2D ligands (MICA and ULBP-2) (Figure S6 in the Supplementary Materials) in response to MHT treatment, whereas no appreciable difference was observed in the expression of ULBP-1 between the different groups (*p* > 0.05) (Figure S6 in the Supplementary Materials).

We additionally found that treatment with mild MHT markedly altered the expression of NK cell inhibitory ligands in U87 cells, including both classical (HLA-I) and non-classical (HLA-E) HLAs (Figure 6). Importantly, for all the examined markers, a non-significant variation in the expression levels was observed in tumor cell groups exposed to AMF or IONCs alone, demonstrating that this re-modulation of NK cell ligands expression is strictly attributable to MHT treatment. 

### 2.5. Mild MHT Sensitizes U87 Cells to NK Cell-Mediated Functions

Previous studies have reported that GBM stem cells exhibit increased expression of Nectin-2, PVR and B7H6 ligands [26,27]. The first two ligands are recognized by NK cells through the DNAM-1 receptor, while B7H6 can promote NK cell activation by interacting with the NKp30 receptor. Overall, the increased expression of these ligands on GBM stem cells has been reported to induce superior receptor-specific activation of NK cells and consequently increase their susceptibility to NK cell-mediated killing [26].

**Figure 7:**
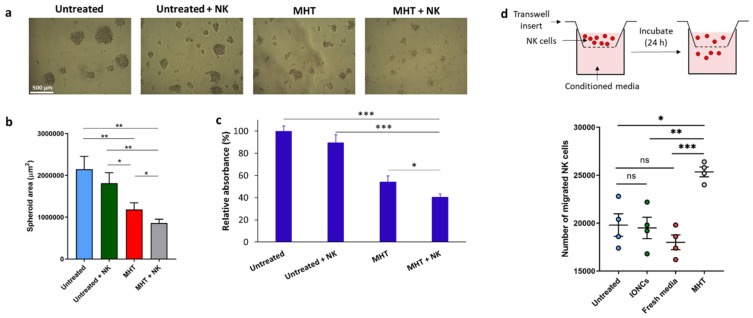
Evaluation of the susceptibility of MHT−treated U87 spheroids to NK cell−mediated killing (**a**–**c**). MHT−treated or untreated U87 cells were cultured for 24 h in serum-free neuronal stem cell medium; following this resting period, IL−2−activated NK cells were added to the culture. At day 7 each culture well was analyzed measuring the area of the spheroids using imageJ software. Representative images per each condition are reported (**a**). Results are presented as mean ± SEM of three experiments (**b**). Cell viability was also evaluated via crystal violet staining (**c**). U87 cells were gently harvested and incubated in conventional adhesion conditions. After 48 h, cell cultures were stained with crystal violet followed by extensive washes (to remove non-adherent cells) and lysis of adherent cells. Relative absorbance at 595 nm was measured with a plate reader. Data are expressed as mean ± SD of three experiments. Chemotaxis of NK cells toward chemokines produced by MHT−treated cells was evaluated using a transwell migration assay (**d**). Conditioned medium collected from U87 cells untreated or treated with IONCs and MHT were added to the lower chambers of transwell plates. Fresh medium was used as a control. NK cells (1 × 10^5^) were added to the upper chamber and cells were incubated for 18 h. Cells in the lower chambers were harvested and counted. Data are shown as mean ± SEM of four independent experiments. Statistical analysis was performed using a one−way ANOVA test (* 0.01 < *p* < 0.05; ** 0.001 < *p* < 0.01; *** *p* < 0.001; n.s. = not significant).

We showed that the expression of CSC-associated ligands can be further upregulated consequently to MHT exposition, and given that their expression has been correlated to increased susceptibility to NK cell-mediated killing, the NK cells killing activity against MHT-treated U87 spheroids was examined. For our purpose U87 spheroids, after MHT treatment, were left to rest for 24 h in serum-free neural stem cell medium. Next, IL-2-activated NK cells were added to pre-treated tumor spheroids, and the spheroids’ growth was monitored. Size analysis of U87 spheroids demonstrated that the combination of MHT and NK cells led to a stronger inhibition of spheroid growth than single treatments (with AMF and no IONCs or with IONCs and no MHT) or NK cells alone (Figure 7a,b). The concomitant analysis of cell viability using a crystal violet staining assay (Figure 7c) provided a further evaluation of the effect of MHT as monotherapy or in combination with NK cells on U87 spheroids. A crystal violet staining assay was carried out after 48 h of incubation of harvested cells at conventional adherent conditions, thus allowing alive cancer cells to adhere to the surface of the plate prior to staining. Non-adherent cells (dying U87 cells and NK cells) were removed following extensive washing, and viability was determined by measuring the absorbance of crystal violet using a spectrophotometer. As shown in Figure 7c, a strong reduction in cell viability was detected in U87 spheroids treated with MHT, indicating that most of the U87 cells had been killed, and this effect was enhanced by NK cells. Indeed, a significant reduction of U87 viability was found in the dual treatment (MHT + NK cells) compared to single treatment with NK cells (*p* < 0.001) or MHT (*p* < 0.001), thus indicating that MHT treatment can sensitize U87 GBM cells to NK cell-mediated killing.

Finally, we investigated if NK cell functionality, in terms of migration and cytotoxicity against U87 cells can be improved by the pre-exposure of tumors cells to mild MHT. By performing a transwell migration assay, we found that conditioned media from MHT-treated U87 cells, enhanced the directional movement of NK cells (Figure 7d), thus providing in vitro evidence that thermal magnetic-triggered therapy could potentially favor NK cell recruitment at the tumor site.

**Figure 8:**
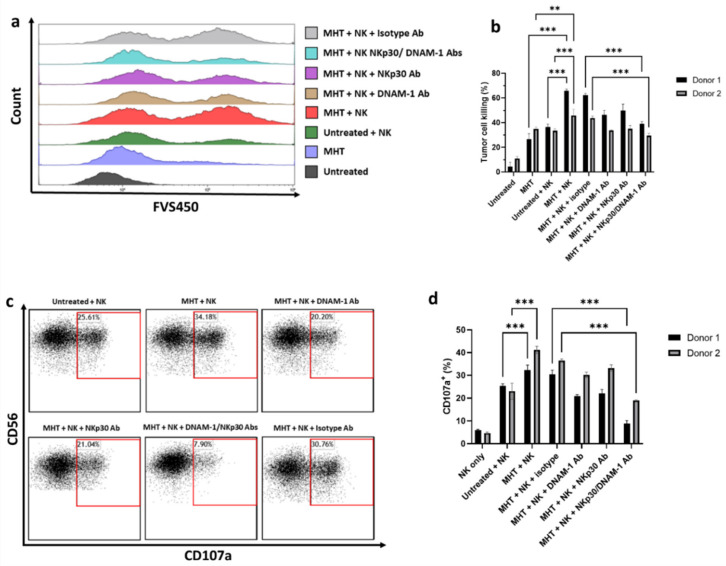
NK cell−mediated killing of U87 cells and degranulation activity. U87 cells were treated with MHT and co−cultured with IL−2−activated NK cells for 4 h at an E:T ratio of 1:1. Tumor cell killing of U87 cells was assessed via flow cytometry determining viability dye-positive (FSV450+) cells in U87 cells. (**a**) Data are shown as representative histograms (**a**) and mean ± SEM (**b**). IL−2−activated NK cells were co−incubated with U87 cells for 4 h at an E:T ratio of 1:1 in the presence of anti−CD107a antibody. The percentage of degranulating CD107a^+^/CD56^+^ NK cells was represented as individual plots (**c**) and mean ± SEM (**d**). Results shown are representative of three experiments using NK cells from two healthy donors. Statistical analysis was performed using a one−way ANOVA test (** 0.001 < *p* < 0.01; *** *p* < 0.001; n.s. = not significant).

The next experiments were addressed at evaluating the impact of MHT treatment on NK cell cytotoxicity against U87 cells. NK cell cytotoxic activity against tumor cells is mainly exerted via the secretion of cytotoxic granules containing granzymes/perforin, resulting in surface exposure of lysosomal-associated proteins that are typically present on the lipid bilayer surrounding lytic granules, such as CD107a. Therefore, we evaluated the membrane expression of CD107a on NK cells as a marker of cytotoxic degranulation. For this, we co-cultured IL-2-activated NK cells, isolated from two healthy donors, with untreated and MHT-treated U87 spheroids, and quantified tumor cell killing and the levels of CD107a in NK cells (Figure 8). Interestingly, pre-treatment of U87 spheroids with MHT resulted in increased sensitivity to NK cell cytotoxic action (*p* < 0.001) (Figure 8a and Figure 2b), and in line with this, MHT pre-treated U87 spheroids induced higher levels of NK cell degranulation compared to untreated U87 spheroids (Figure 8c,d). The enhanced NK cell reactivity against MHT-pretreated GBM spheroids was also observed in terms of IFN-γ production (Figure S7 in the Supplementary Materials). Finally, we demonstrated that the killing of MHT pre-treated U87 GBM spheroids involves both DNAM-1 and NKp30 receptors, since double blocking treatment with anti-DNAM-1 and anti-NKp30 antibodies caused a strong inhibition of killing and degranulation of IL-2-activated NK cells, compared with single blocking of DNAM-1 or NKp30 (*p* < 0.001). 

## 3. Discussion

The exploitation of ICD to revert the immunosuppressive TME into a more immunostimulatory one, and trigger an effector antitumor immune response sustained by both innate and adaptive effector cells against stressed/dying cancer cells is emerging as a novel strategy for the treatment of tumors that are classified as immunologically “cold”.

This is particular interesting for GBM since recent evidence suggests a predominant role of innate immunity in brain tumor surveillance. Indeed, GBM shows a “non-conventional” immunological profile and unlike other tumors, such as melanoma and breast cancer, NK cells represent the most abundant tumor-infiltrating lymphocytes and up to half of GBM mass is constituted by resident microglia and circulating blood monocytes/macrophages [23,25].

Although MHT-induced anticancer responses have been reported in several cancers, including melanoma, breast cancer and colon carcinoma, little is known regarding MHT as an ICD inducer in GBM and its impact on the interactions between cancer cells and effector innate immune cells [54,55]. Our results demonstrate that mild MHT treatment can be exploited as the external activation mechanism to induce ICD and re-modulate the immunogenic profile of U87 GBM cells, thus rendering them more susceptible to the antitumoral action of innate immune effectors, such as macrophages and NK cells. The results of the present study demonstrated that treatment with mild MHT was cytotoxic in U87 cells, with 30% reduction in cell viability, and that it can re-shape the immunological features of these tumor cells, thus facilitating their recognition by innate effectors, including macrophages and NK cells. 

MHT treatment, mediated by IONC-GA-PEG, induced the upregulation of several immunogenic molecules (CRT, HSP70, HSP90 and HMGB-1) and downregulated the expression of immunological “breaks” (CD47 and PD-L1) that can promote immune evasion. Differential expression of DAMPs resulted in a faster recognition and phagocytosis of U87 cells by macrophages in vitro. Upon 24 h of co-culture, macrophages incubated in the presence of U87 cells pre-treated with mild MHT displayed an activated profile with increased surface levels of CD86 and MHC-II. 

Once we showed that, in line with other previous studies conducted with other types of cancer cells, MHT can induce ICD in U87 GBM cells, we decided to focus our attention on the potential role that NK cells can play in mediating MHT-triggered immune responses.

First, we showed that treatment with mild MHT induces increased expression of DNAM-1 and NKp30 ligands and downregulation of MHC molecules in U87 human GBM cells, thereby potentially enhancing their susceptibility to NK cell-mediated killing. Consequently, we found that pre-treatment of GBM cells with MHT positively reflected on the functionality of IL-2-activated NK cells in terms of degranulation and IFN-γ release.

Importantly, a DNAM-1 and NKp30 blockade reduced the lysis of cancer target cells, and together the two blocking antibodies displayed a synergistic effect that abrogates NK cell-mediated killing of U87 cells pre-sensitized via treatment with MHT. This result is consistent with the reduction in NK cell degranulation upon pre-treatment with anti-DNAM-1 and NKp30 blocking antibodies of MHT-treated GBM cells. Together, these findings provide clear evidence that mild MHT treatment can not only induce adaptive immunity by serving as an ICD inducer for the activation of APCs, but can also sensitize U87 GBM cells to NK cell-mediated killing.

Even if new ablative physical treatments have been recently approved for clinical use in patients with GBM, including tumor treating fields (TTFields) and laser interstitial thermal therapy (LITT), important challenges are still associated with TTFields and LITT, which include the elevated costs associated with these medications and the limited applicability to only certain subtypes of GBMs depending on their size, morphology and localization [67,68,69]. Moreover, the ability of TTFields and LITT to serve as ICD inducers still needs to be proven. The latter is particularly true in the case of TTFields, which induces cell death mostly via apoptosis, as it has been demonstrated to fail at triggering APC activation and, instead, promotes immune tolerance [70]. Furthermore, unlike laser-based treatments, MHT provides a remote activation modality with no tissue penetration depth problems.

All of the above, together with the demonstrated ability to target CSCs, makes MHT one of the most valid options as an ICD inducer among the various approved ablative treatments [71]. 

## 4. Conclusions

In summary, we showed that mild MHT with magnetic nanoparticles can be applied not only as an “in situ vaccination” strategy, but can also be explored as a novel approach to enhance the antitumoral activity of NK cells in GBM. This occurs by re-modulating the immunological profile of target cells via induction of stress-associated molecules and downregulation of inhibitory ligands. These results could open new therapeutic possibilities for MHT, providing a rationale for novel combinatorial treatments based on MHT and NK cell therapies, with the potential to simultaneously ensure TIME re-modulation and the generation of a NK cell-sustained effector response. However, at the same time, we also observed slight reduction in the expression of some NKG2D ligands on the surface of U87 cells that were exposed to mild MHT. Thus, future in vitro and in vivo studies are needed to further explore the association of mild MHT treatment with the expression of NK ligands. Thereby, it will be possible to define if this concept can be generalized to GBM or even extended to other kinds of tumors. Furthermore, a comprehensive elucidation of the molecular mechanisms behind the effects of mild MHT on the tumor-NK cell interaction could enable a full exploitation of NK cell-based therapeutic potential.

## 5. Experimental Section

### 5.1. Reagents

U87 and THP-1 cell lines were purchased from American Type Culture Collection (ATCC, Manassas, VA, USA). All chemicals were obtained from Sigma-Aldrich (St. Louis, MO, USA). Annexin-FITC/PI kit was obtained from Miltenyi Biotec (Bergisch Gladbach, Germany). Antibodies for E-cadherin and Vimentin were purchased from Santa Cruz Biotechnology (Dallas, TX, USA). Antibodies against CRT, HSP70 and HSP90 were obtained from Enzo Life Sciences (Milan, Italy). Antibodies for CD133, CD47, CD14, HLA-I (ABC), HLA-E, HLA-DR, CD11b, CD86 and PD-L1 were purchased from Biolegend (Milan, Italy). Antibodies against ULBP-1, ULBP-2 and ULBP-3 were purchased from R&D Systems (Minneapolis, MN, USA). Anti-CD16 antibody was obtained from Miltenyi Biotec (Bergisch Gladbach, Germany). Anti-CD107a antibody was purchased from Thermo Fisher Scientific (Milan, Italy). Anti-CD56 antibody was purchased from Beckman Coulter (Milan, Italy). Fixable viability staining 450 (FVS450) was provided by BD Biosciences (San Jose, CA, US). Carboxyfluorescein diacetate succinimidyl ester (CFSE) was purchased from Thermo Fisher Scientific. Antibodies against Nectin-2, PVR and B7H6 were obtained from R&D Systems. HMGB1 ELISA kit was obtained from Tecan (Männedorf, Switzerland). An IFN-γ ELISA kit was purchased from Invitrogen (Milan, Italy).

### 5.2. Synthesis of the IONCs

The synthesis of the IONCs was conducted according to the procedure reported in the patent [61]. Briefly, to prepare the IONCs, a solvothermal method was used and it requires three steps: (i) providing a solution comprising oleic acid, hexadecylamine and 1-octanol; (ii) adding a solution of iron pentacarbonyl and benzaldehyde; (iii) transferring the reaction mixture to an autoclave vessel; and (iv) heating the autoclave to a temperature of 200 °C for 6 h.

### 5.3. Synthesis of Gallic Acid PEG (GA-PEG) Ligand

The synthesis of GA-GEG was that reported by Guardia et al. with some modifications [62]. Briefly, 5 g poly(ethylene glycol) (PEG, 3.3 mmol, Mw = 1500 kDa) was dissolved in 500 mL tetrahydrofuran (THF) through sonication for 1 h. Then, 280 mg of Gallic acid (GA, 1.64 mmol) was dissolved in 10 mL THF, and separately 20 mg dimethyl amino pyridine (DMAP, 0.16 mmol) was dissolved in 10 mL THF in a flask under magnetic stirring at room temperature. Next, 1.72 g N.N’-dicyclohexylcarbodiimide (DCC, 8.3 mmol) dissolved in 20 mL of THF was dropwise added within 1 h to the solution of PEG/GA/DMAP under energic magnetic stirring. The mixture was then stirred at room temperature for additional 48 h, and THF and DMAP were removed under reduced pressure (300 mbar). The crude product (GA-PEG) was completely dried and then dissolved in 40 mL de-ionized water and the pH adjusted to 2 to precipitate hydrolyzed DCC. After 1 h, the solution was filtered with a paper filter in a Buchner funnel and GA-PEG was extracted from the aqueous phase with 200 mL chloroform. This step was repeated 5 times. After removal of chloroform at reduced (350 mbar) pressure, GA-PEG was dried in the vacuum stove overnight at 44 °C. GA-PEG was dissolved in 20 mL of dichloromethane (DCM) and added dropwise in 200 mL of cold diethyl ether. After this, the precipitate was filtered with a Buchner funnel and dried in the vacuum stove overnight at 40 °C. Prior to determination of the yield, a 0.05M solution of GA-PEG in chloroform was prepared and further used for the water transfer of the IONCs. The yield was determined through ^1^H-NRM and was found to be 11%.

### 5.4. Ligand Exchange and Water Transfer of Cubic Iron Oxide Nanoparticles (IONCs-GA-PEG)

Briefly, 4 mL of a chloroform solution of the IONCs ([Fe] = 1mg/mL) was added to a 10.8 mL GA-PEG solution (0.05 M) to provide 500 ligand molecules per nm^2^ of nanoparticle surface. Subsequently, 1.08 mL of triethylamine (TEA) was added. The solution was stirred in an orbital shaker overnight at 3000 rpm. The sample was extracted with 5 mL of toluene, 10 mL of Milli-Q water and small amounts of acetone. IONCs spontaneously phase transferred from the organic to the aqueous phase. Any trace of acetone was removed from the aqueous phase by bubbling nitrogen for at least 30 min. The sample was concentrated through rotavaporation and it was further purified via dialysis overnight and at room temperature against Milli-Q water (5 L) to remove the unreacted GA-PEG. Cellulose membrane tubes with a molecular weight cut-off (MWCO) of 50 kDa were chosen for the dialysis. This step was repeated twice. Finally, the resulting GA-PEG-coated IONC sample was concentrated via ultrafiltration using an Amicon centrifugal filter (MWCO of 100 kDa, Merck Millipore, Milan, Italy) and analyzed via dynamic light scattering (DLS) (Zetasizer Nano ZS90, Malvern Instruments Ltd, Worcestershire, UK) and transmission electron microscopy (TEM).

### 5.5. SAR Measurements

The calorimetric measurements to quantify the specific absorption rate (SAR) value of the IONCs were conducted using a commercially available magnetic nano-heating device (DM100 Series, nanoscale Biomagnetics, Zaragoza, Spain). The aqueous solution of IONCs was exposed to an alternating magnetic field with amplitudes of 12, 16 or 24 kA/m and frequencies of 100 or 300 kHz. SAR values were calculated using the following equation:

SAR = (C/m)(dT/dt), (1)

where C is the specific heat capacity of the colloid (C_water_ = 4.18 J g^−1^ C^−1^), dT/dt is the initial slope of the time-dependent temperature curve, and m is mass of magnetic material (g/L) in the suspension. To calculate the parameter dT/dt, temperature data points collected within the first 60 s were used to obtain the slope of the curve deriving from the linear fitting of these points.

### 5.6. Cell Lines and Cell Cultures

U87 cells were cultured in Dulbecco’s modified Eagle’s medium (DMEM; Sigma-Aldrich high glucose with 4500 mg/L of glucose) and supplemented with 10% heat-inactivated fetal bovine serum (FBS; Sigma-Aldrich), 2 mM L-glutamine (Sigma-Aldrich) and 100 U/mL penicillin/streptomycin (Sigma-Aldrich) at 37 °C in a humidified 95% air and 5% CO_2_ atmosphere.

U87 spheroids were cultured in serum-free neural stem cell culture medium composed of DMEM/F-12 (Sigma-Aldrich) supplemented with B-27 without vitamin A, 20 ng/mL of both epidermal growth factors (EGF; PeproTech, Rocky Hill, NJ, USA), 20 ng/mL basic fibroblast growth factor (FGF; PeproTech), 5 μg/mL heparin and 1% penicillin/streptomycin into ultra-low attachment 96-well microplates with flat bottom (Corning, Milan, Italy).

THP-1 cells were grown in RPMI-1640 (Sigma-Aldrich) supplemented with 10% heat-inactivated FBS, 2 mM L-glutamine, 100 U/mL penicillin/streptomycin and 50 μM β-mercaptoethanol (Sigma-Aldrich). THP-1 monocytes were differentiated into macrophages via 48 h of incubation with 100 ng/mL phorbol 12-myristate 13-acetate (PMA, Sigma-Aldrich) followed by 48 h of incubation in RPMI 1640 medium. Differentiation was confirmed by evaluating the expression of CD14 using BD FACSAria III flow cytometry (BD Biosciences) (Figure S8 in the Supplementary Materials).

### 5.7. Cell Treatment with MHT

Magnetic hyperthermia studies on cells were performed at 182 kHz and 16 kA/m field conditions for exposure to three consecutive cycles of MHT separated by a 5-min break under AMF of 30 min each and at a IONCs concentration of 2 g_Fe_/L. In detail, adherent U87 cells were detached with trypsin and counted using a hemocytometer. Afterwards, 1.5 × 10^6^ cells were resuspended in 150 μL of medium containing IONCs within a small glass vial. Then, cells were exposed to AMF. Immediately after treatment, the cells were washed with PBS and resuspended in serum-free neural stem cell culture medium. 

### 5.8. Cell Viability Assay

To measure cell viability, cells were plated in 96-well plates (5 × 10^3^ cells per well) and allowed to grow for 24 h before treatment. Cells were then exposed to different treatments, such as only magnetic field without IONCs (AMF), IONCs with no exposure to AMF (IONCs) and IONCs and MHT exposure (MHT). After incubation for 24 and 48 h, cell viability was evaluated using a CCK-8 assay kit (Abcam, Milan, Italy) according to the manufacturer’s instructions.

### 5.9. Annexin V-FITC/Propidium Iodide Assay

Cell death was determined using an Annexin V-FITC/Propidium Iodide (PI) kit (MACS, Miltenyi Biotech, Bergisch Gladbach, Germany) according to the manufacturer’s instructions. Briefly, after treatment, cells were washed with 1× binding buffer and then 10^6^ cells from each group were stained with AnnexV-FITC for 15 min in the dark, followed by PI staining. The stained cells were analyzed using flow cytometry (BD FACSAria III). The results were expressed as percentage of living (AnnexV^−^/PI^−^), primary necrotic (AnnexV^−^/PI^+^), apoptotic (AnnexV^+^/PI^−^) and secondary necrotic cells (AnnexV^+^/PI^+^). Data were analyzed using FCS express 7 research edition (De Novo Software, Pasadena, CA, USA).

### 5.10. Flow Cytometry Analysis of Surface Markers on U87 Cells

For flow cytometry analysis, U87 cells were collected using TrypLE Express Enzyme (Gibco, Basel, Switzerland) or Gentle Cell Dissociation Reagent (Stem Cell Technologies, Milan, Italy). Cells were then transferred into 15 mL tubes (Sigma-Aldrich) and washed with FACS buffer (ice-cold PBS with 2% FBS). Then 1 × 10^5^ cells were resuspended in 100 µL of FACS buffer containing antibodies and incubated for 30 min on ice. All subsequent incubation steps were carried out in the dark. Cells were washed with 1 mL of FACS buffer and centrifuged at 300× *g* for 5 min at 4 °C. Then, the cells were resuspended in FACS buffer and analyzed using a BD FACSAria III. Unspecific background of individual channels was determined with isotype controls, and color compensation was done on single color-stained samples. FACS plots were generated with FCS express 7 software (DeNovo Software).

### 5.11. Cell Cycle Analysis

Cells were harvested and washed twice with ice-cold 1 × PBS. Then, 1 × 10^6^ cell pellets were resuspended in 4.0 mL ice-cold 70% ethanol by adding with a Pasteur pipette on a vortex. After 2 h of incubation at 4 °C, cells were pelleted via centrifugation at 1000× *g* for 5 min and washed twice with 1 × PBS. Then, cells were resuspended with 300 µL of DAPI (1µg/mL, Sigma-Aldrich)/Triton X-100 (0.1%, Sigma-Aldrich) solution and incubated for 30 min at room temperature protected from the light. The cells were analyzed for DNA content via flow cytometry (BD FACSAria III, BD Biosciences, Franklin Lakes, NJ, USA). The relative proportions of cells in the G0-G1 phase (2n), S phase (>2n but <4n) and G2/M phase (4n) of the cell cycle were determined using FCS express 7 software (De Novo Software).

### 5.12. Transwell Migration Assay

Transwell migration assay of U87 cells was performed using a 24-well transwell plate containing inserts with polycarbonate membrane with pores of 8 µm (Corning). Then, 1 × 10^5^ cells resuspended in 100 μL of serum-free medium were placed in the upper insert and the outer chamber was filled with 600 μL of medium containing 10% FBS. After incubation for 18 h, the inserts were transferred to a new plate containing pre-warmed (37 °C) trypsin-EDTA. Medium with 10% FBS was added to inactivate trypsin. Cells that had migrated through the pores into the lower chamber were detected by measuring their cellular ATP content using a CellTiter-Glo 2.0 reagent. The luminescence was measured at 548 nm using a microplate reader (Tecan Spark, Milan, Italy).

Chemotaxis of NK cells was tested using a transwell assay. Briefly, lower chambers of 24-well transwell plates (3.0 μm pore size, Corning) were filled with 600 μL of conditioned medium collected from untreated cells or cells exposed to different treatments. Then, 600 μL of fresh medium was used as a control. Approximately 1 × 10^5^ of NK cells was added in 100 μL of serum-free medium (RPMI containing 25 mM HEPES and 0.5% BSA) in the upper chamber, and cells were incubated for 18 h. Cells were harvested via centrifugation (300× *g* for 5 min at 4 °C), resuspended in 100 μL PBS, stained with trypan blue and counted using a hemocytometer.

### 5.13. Colony Formation Assay

Immediately after treatment, cells were grown under adherent conventional conditions with complete medium in 6-well plates (500 cells per well) for 14 days. Then, the cells were washed with 1× Phosphate-buffered saline (PBS, Sigma-Aldrich), fixed with ice-cold methanol for 10 min at room temperature and subsequently stained with 0.5% crystal violet (Sigma-Aldrich) for 15 min. After washing with water, the stained cells were photographed, and cell growth was quantified by dissolving crystal violet in SDS (2%) and measuring the absorbance at 595 nm (Multiskan, Thermo Fisher Scientific).

### 5.14. In Vitro Phagocytosis, Killing and Activation of THP-1-Derived Macrophages

Dissociated cancer cells were washed twice and resuspended at 1 × 10^6^ cells/mL in 1 mL of 1 × PBS containing CFSE (5 µM). After incubation for 10 min in a 37 °C water bath, the cells were washed with ice-cold 1× PBS to remove the excess of CFSE.

Stained cells were resuspended in serum-free DMEM at a concentration of 5 × 10^4^ cells/mL and transferred into a 24-well plate containing THP-1-derived macrophages (5 × 10^4^ cells/mL), prepared as previously described, for a final effector: target ratio (E:T ratio) of 1:1. Cells were co-cultured for two hours, then harvested, stained with anti-human CD11b for 30 min at 4 °C and washed twice with 2% FBS in 1× PBS.

Phagocytosis was determined via flow cytometry and data analysis performed using FCS express 7 software (De Novo Software). The phagocytic index was calculated as the percentage of CD11b^+^/CFSE^+^ macrophages. The killing of cancer cells by THP-1-derived macrophages was evaluated via crystal violet staining following 48 h of incubation and quantified by measuring the absorbance at 595 nm with a plate reader (Multiskan, Thermo Fisher Scientific). For stimulation with TLR-agonist, a Class C CpG-ODN (ODN 2395) was used at a concentration of 1 µg/mL. The killing was calculated using the following formula: killing (%) = [(Absorbance_(treated tumor cells + Macrophages)_ − Absorbance_(Macrophages)_)/(Absorbance_(Treated tumor cells + Macrophages)_ –Absorbance_(Macrophages)_)] × 100.

The expression of cell surface activation markers on THP-1 cells was analyzed via flow cytometry. THP-1 macrophages were detached from culture flasks by gentle scraping and incubated for 30 min at 4 °C in PBS with 2% FBS containing anti-CD11b, anti-CD86 and anti-HLA-DR antibodies. The expression of CD86 and HLA-DR on CD11b cells was analyzed using BD FACSAria III (BD Biosciences), and data were processed using FCS express 7 software (De Novo Software). 

### 5.15. HMGB-1 ELISA

After exposure to different treatments, U87 cells were seeded in 24-well plates and incubated for 24 h. Supernatants were collected for high mobility group box 1 (HMGB-1) detection using an ELISA kit (Tecan), according to the manufacturer’s instructions.

### 5.16. NK Cell Preparation

NK cells were isolated from peripheral blood using the Ficoll‒Paque density gradient to obtain peripheral blood mononuclear cells (PBMCs) followed by purification using an NK cell isolation kit (RosetteSep kit, StemCell Biotechnologies, Milan, Italy). After isolation, NK cells were cultured in round-bottom 96-well plates in 200 µL of complete RPMI 1640 medium (Sigma-Aldrich) supplemented with 10% fetal calf serum (FCS), 2 mM L-glutamine, 100IU/mL rhIL-2 and 100 U/mL penicillin/streptomycin and containing 10^5^ irradiated PBMCs and a 5 × 10^3^ 721.221 lymphoblastoid cell line transfected with HLA-G. CD3^−^CD56^+^ clones were obtained by culturing highly purified CD3^−^CD19^−^CD14^−^ NK cells under limiting dilution conditions as previously reported [72].

### 5.17. NK Cell Functional Assays

Untreated and treated cells were cultured in serum-free neural stem cell culture medium at 5 × 10^3^ cells/well in flat-bottom ultra-low-attachment 96-well plates (Corning). At 24 h post-treatment, IL-2-activated NK cells (100 U/mL) were added to the U87 cells at 2.5 × 10^4^ cells/mL. Generation of spheroids was monitored until day 7 and the spheroid area was analyzed in each culture well by acquiring images with a default microscope with 4× objective and analyzing them with imageJ software (ImageJ 1.53f, National Institutes of Health, Bethesda, MD, USA). The supernatants were collected to measure the release of IFN-γ using an ELISA kit according to manufacturer’s instructions (Biolegend).

Crystal violet cytotoxicity assay was also performed to determine the killing activity of NK cells. Briefly, spheroids at day 5, alone or co-cultured with NK cells, were transferred in conventional adherent plates and after 48 h were stained with crystal violet as previously described. After extensive washing, adherent cells were solubilized and the amount of crystal violet proportional to the number of living cells was estimated with a plate reader (Multiskan, Thermo Fisher Scientific) measuring the absorbance at 595 nm.

Degranulation and viability were assessed via flow cytometry on NK cells and U87 target cells, respectively. Briefly, U87 cells exposed or not to treatment with MHT were incubated for 24 h in serum-free neural stem cell culture medium. Then, U87 cells were co-cultured with IL-2-activated NK cells (100 U/mL) at an E:T ratio of 1:1 (1 × 10^5^ cells: 1 × 10^5^ cells) in the presence of eFluor660-conjugated anti-CD107a. Before co-culture with target cells, NK cells were either left untreated or blocked for 30 min with anti-DNAM-1 antibody (10µg/mL, Miltenyi Biotec) or anti-NKp30 antibody (10µg/mL, Miltenyi Biotec). After 1 h of culture, monensin (BD golgi stop, BD Biosciences) was added and cells were cultured for a further 3 h before staining for CD56 and viability with FVS450.

### 5.18. Statistical Analysis

Graphpad prismTM 7.0 software was used for analyzing and graphing the data. Statistical significance was determined using an unpaired student’s *t* test or one-way ANOVA. Differences were considered statistically significant if *p* < 0.05 (* *p* < 0.05; ** *p*< 0.01; *** *p* < 0.001; and n.s., not significant). Data were expressed as mean ± SD or mean ± SEM.

## Data Availability

The data presented in this study are available within the article and Supplementary Materials.

## Supplementary Materials

The following are available online at https://www.mdpi.com/article/10.3390/pharmaceutics13101668/s1. Figure S1: Histogram of size distribution and Gaussian fitting curve of IONC-GA-PEG analyzed by TEM, Figure S2: SAR values of IONC-GA-PEG at frequencies of 100 (red triangles) and 300 (blue triangles) kHz, and field amplitudes of 12, 16 and 24 kA/m, Figure S3: Evaluation of cell viability of U87 cells exposed to various treatment conditions, such as no treatment (untreated group), AMF, IONCs at a concentration of 2 g_Fe_/L and MHT (2 g_Fe_/L of IONCs + AMF). Cell viability was determined by CCK-8 assay at 24 and 48 h post-treatment. Statistical analysis was conducted via two-tailed unpaired student’s *t* test. *** *p* < 0.001; n.s.= not significant, Figure S4: Flow cytometry analysis of cell cycle in U87 cells after 24 h post-treatment with AMF, IONCs or MHT compared to untreated cells. Representative figures of cell cycle distribution (SubG1. G0/G1, S, and G2/M) showing accumulation of MHT-treated cells in S and G2/M phases, Figure S5: Release of HMGB1 in response to MHT treatment was evaluated by ELISA (a). The expression of PD-L1 on the surface of untreated cells or U87 cells exposed to different treatment (AMF, IONCs and MHT) was evaluated by flow cytometry (b,c). Data are shown as expressed as average fold change (mean ± SD) of the mean fluorescence intensity (MFI) of three independent experiments (*n* = 3) and as representative histograms. Statistical analysis was conducted via two-tailed unpaired student’s *t* test (* 0.01 < *p* < 0.05; *** *p* < 0.001; n.s. = not significant), Figure S6. Flow cytometric evaluation of changes in surface expression of MICA, ULBP-1 and B7H6 in U87 cells exposed to different treatments (AMF, IONCs and MHT) compared to untreated cells at 24 h post-treatment. Data are shown as representative histograms and expressed as average fold change (mean ± SD) of the mean fluorescence intensity (MFI) of three independent experiments (*n* = 3). Statistical analysis was conducted via two-tailed unpaired student’s *t* test (* 0.01 < *p* < 0.05; *** *p* < 0.001; n.s. = not significant), Figure S7: Analysis of IFN-γ secretion by IL-2-activated NK cells co-cultured with untreated or treated U87 GBM cells. Only NK cells and, untreated and MHT-treated U87 (MHT) cells alone were included as control groups. Data are shown as mean ± SEM of three experiments. Statistical analysis was performed using a one-way ANOVA test (*** *p* < 0.001), Figure S8: Evaluation of CD14 expression on THP-1 cells before and after macrophage differentiation by flow cytometry.
