# Supplementary material for: Elucidating the Innate Immunological Effects of Mild Magnetic Hyperthermia on U87 Human Glioblastoma Cells: An In Vitro Study
Source: Pharmaceutics. 2021 Oct 12;13(10):1668. doi: 10.3390/pharmaceutics13101668 (PMC8537446; doi:10.3390/pharmaceutics13101668)
Supplement: Supplementary file 1 [file pharmaceutics-13-01668-s001.zip › pharmaceutics-1351887-supplementary.pdf]

pharmaceutics

MDPI

# Supplementary Materials: Elucidating the Innate Immunological Effects of Mild Magnetic Hyperthermia on U87 Human Glioblastoma Cells: An In Vitro Study

**Stefano Persano, Francesco Vicini, Alessandro Poggi, Jordi Leonardo Castrillo Fernandez, Giusy Maria Rita Rizzo, Helena Gavilán, Niccolo Silvestri [1] and Teresa Pellegrino**

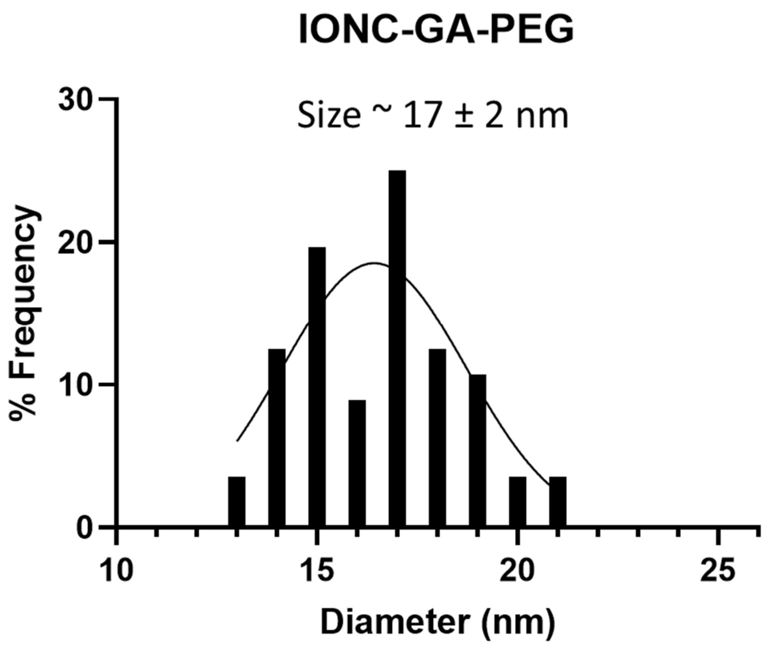


**Figure S1.** Histogram of size distribution and Gaussian fitting curve of IONC-GA-PEG analyzed by TEM.

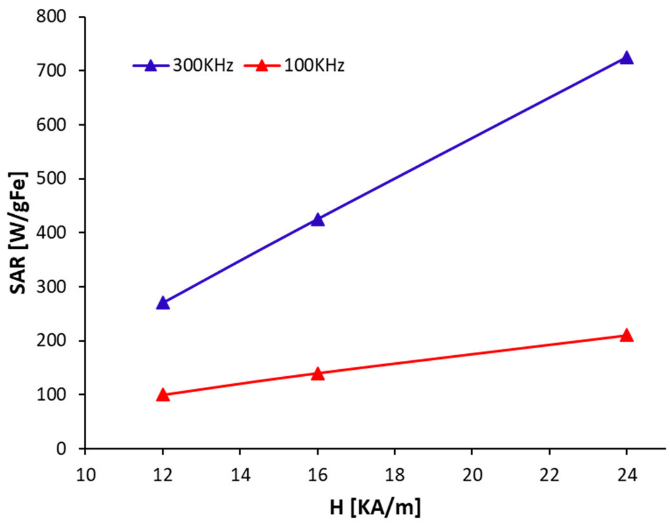


**Figure S2.** SAR values of IONC-GA-PEG at frequencies of 100 (red triangles) and 300 (blue triangles) kHz, and field amplitudes of 12, 16 and 24 kA/m.

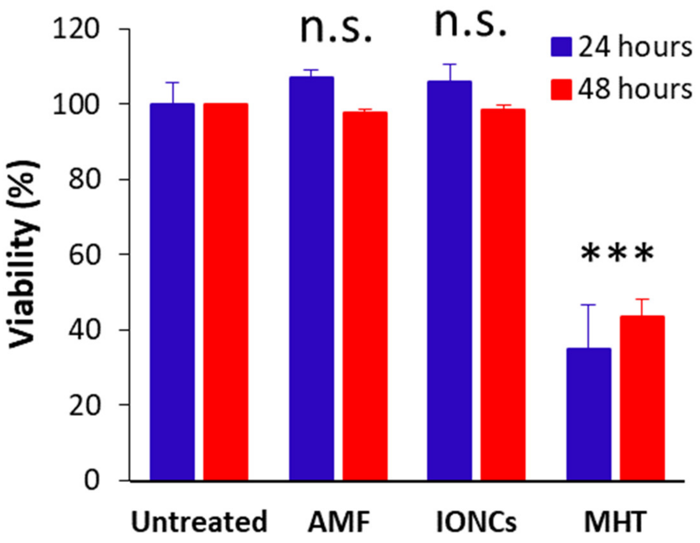


**Figure S3.** Evaluation of cell viability of U87 cells exposed to various treatment conditions, such as no treatment (untreated group), AMF, IONCs at a concentration of 2 $g_{Fe}$/L and MHT (2 $g_{Fe}$/L of IONCs + AMF). Cell viability was determined by CCK-8 assay at 24 and 48 hours post-treatment. Statistical analysis was conducted via two-tailed unpaired student's t test (*** $p < 0.001$; n.s.= not significant).

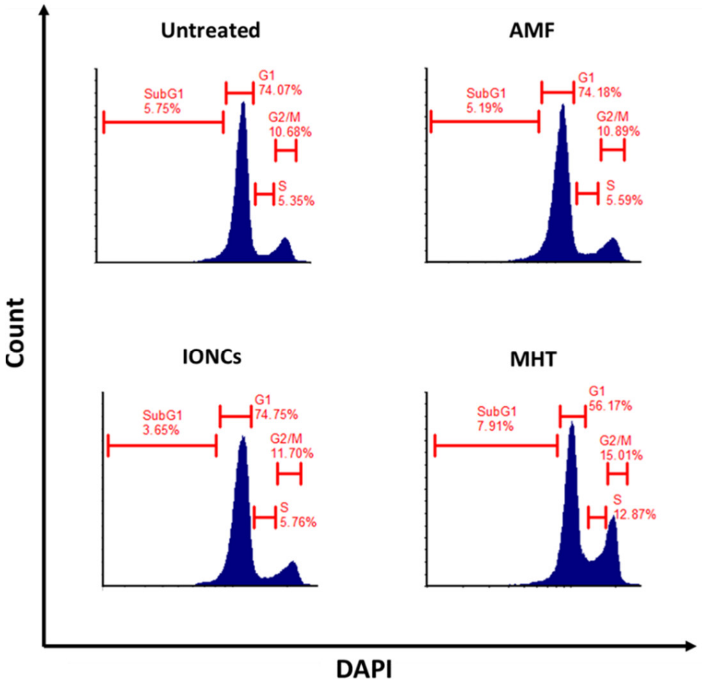


**Figure S4.** Flow cytometry analysis of cell cycle in U87 cells after 24 hours post-treatment with AMF, IONCs or MHT compared to untreated cells. Representative figures of cell cycle distribution (SubG1. G0/G1, S, and G2/M) showing accumulation of MHT-treated cells in S and G2/M phases.

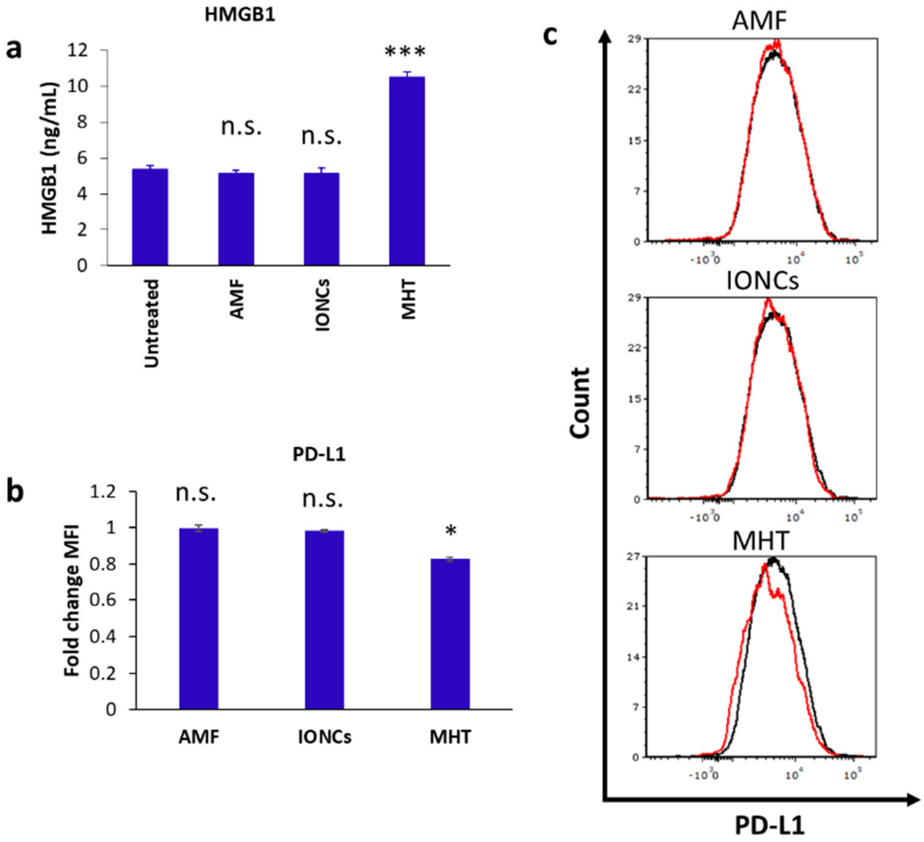


**Figure S5.** Release of HMGB1 in response to MHT treatment was evaluated by ELISA (**a**). The expression of PD-L1 on the surface of untreated cells or U87 cells exposed to different treatment (AMF, IONCs and MHT) was evaluated by flow cytometry (**b**,**c**). Data are shown as expressed as average fold change (mean ± SD) of the mean fluorescence intensity (MFI) of three independent experiments ($n = 3$) and as representative histograms. Statistical analysis was conducted via two-tailed unpaired student's t test (* $0.01 < p < 0.05$; *** $p < 0.001$; n.s. = not significant).

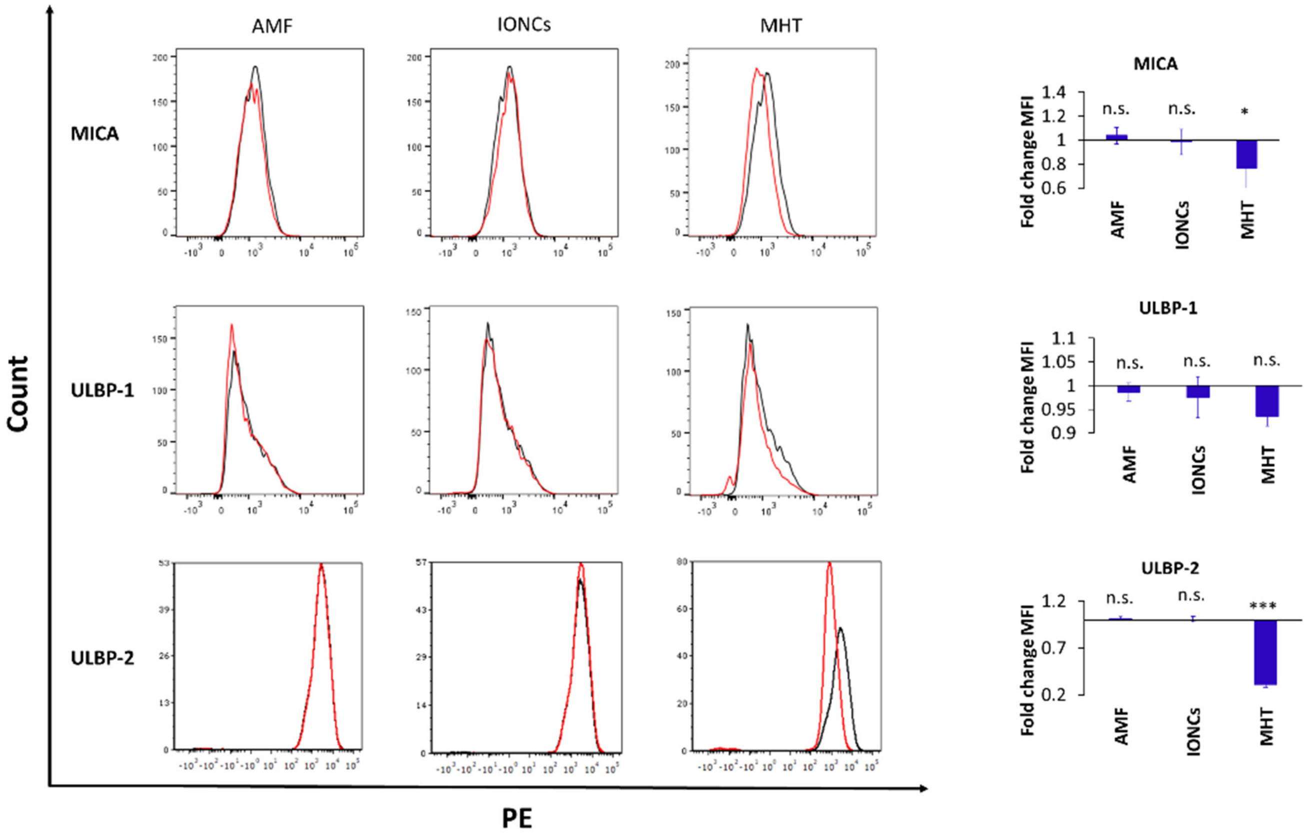


**Figure S6.** Flow cytometric evaluation of changes in surface expression of MICA, ULBP-1 and B7H6 in U87 cells exposed to different treatments (AMF, IONCs and MHT) compared to untreated cells at 24 hours post-treatment. Data are shown as representative histograms and expressed as average fold change (mean ± SD) of the mean fluorescence intensity (MFI) of three independent experiments ($n = 3$). Statistical analysis was conducted via two-tailed unpaired student's t test (* $0.01 < p < 0.05$; *** $p < 0.001$; n.s.= not significant).

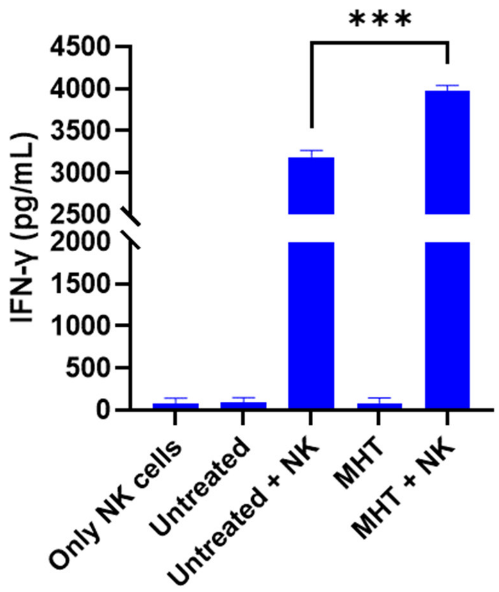


**Figure S7.** Analysis of IFN-γ secretion by IL-2-activated NK cells co-cultured with untreated or treated U87 GBM cells. Only NK cells and, untreated and MHT-treated U87 (MHT) cells alone were

included as control groups. Data are shown as mean ± SEM of three experiments. Statistical analysis was performed using a one-way ANOVA test (*** $p < 0.001$).

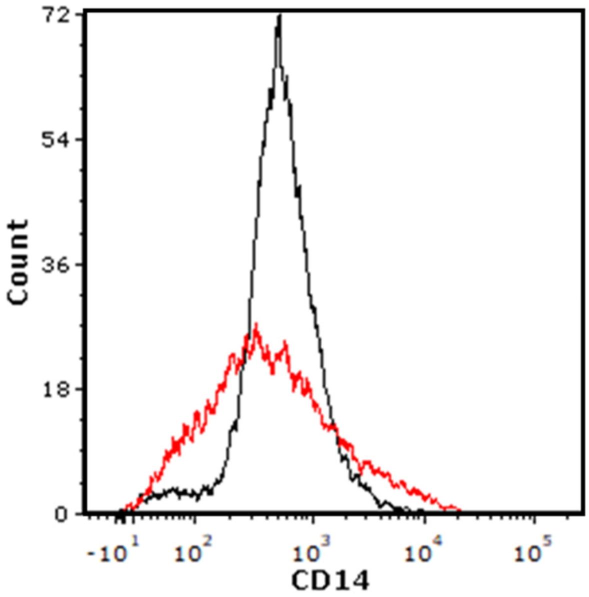


**Figure S8.** Evaluation of CD14 expression on THP-1 cells before and after macrophage differentiation by flow cytometry.
